# Selective Inactivation of Fibroblast Growth Factor 22 (FGF22) in CA3 Pyramidal Neurons Impairs Local Synaptogenesis and Affective Behavior Without Affecting Dentate Neurogenesis

**DOI:** 10.3389/fnsyn.2017.00017

**Published:** 2017-12-19

**Authors:** Akiko Terauchi, Elizabeth Gavin, Julia Wilson, Hisashi Umemori

**Affiliations:** F.M. Kirby Neurobiology Center, Department of Neurology, Boston Children’s Hospital, Harvard Medical School, Harvard University, Boston, MA, United States

**Keywords:** hippocampus, synaptogenesis, neurogenesis, fibroblast growth factor, conditional knockout mice, CA3, depression

## Abstract

Various growth factors regulate synapse development and neurogenesis, and are essential for brain function. Changes in growth factor signaling are implicated in many neuropsychiatric disorders such as depression, autism and epilepsy. We have previously identified that fibroblast growth factor 22 (FGF22) is critical for excitatory synapse formation in several brain regions including the hippocampus. Mice with a genetic deletion of FGF22 (FGF22 null mice) have fewer excitatory synapses in the hippocampus. We have further found that as a behavioral consequence, FGF22 null mice show a depression-like behavior phenotype such as increased passive stress-coping behavior and anhedonia, without any changes in motor, anxiety, or social cognitive tests, suggesting that FGF22 is specifically important for affective behavior. Thus, addressing the precise roles of FGF22 in the brain will help understand how synaptogenic growth factors regulate affective behavior. In the hippocampus, FGF22 is expressed mainly by CA3 pyramidal neurons, but also by a subset of dentate granule cells. We find that in addition to synapse formation, FGF22 also contributes to neurogenesis in the dentate gyrus: FGF22 null mice show decreased dentate neurogenesis. To understand the cell type-specific roles of FGF22, we generated and analyzed CA3-specific FGF22 knockout mice (FGF22-CA3KO). We show that FGF22-CA3KO mice have reduced excitatory synapses on CA3 pyramidal neurons, but do not show changes in dentate neurogenesis. Behaviorally, FGF22-CA3KO mice still show increased immobility and decreased latency to float in the forced swim test and decreased preference for sucrose in the sucrose preference test, which are suggestive of a depressive-like phenotype similar to FGF22 null mice. These results demonstrate that: (i) CA3-derived FGF22 serves as a target-derived excitatory synaptic organizer in CA3 *in vivo*; (ii) FGF22 plays important roles in dentate neurogenesis, but CA3-derived FGF22 is not involved in neurogenesis; and (iii) a depression-like phenotype can result from FGF22 inactivation selectively in CA3 pyramidal neurons. Our results link the role of CA3-derived FGF22 in synapse development, and not in neurogenesis, to affective behavior.

## Introduction

Growth factor signaling is implicated in many aspects of brain development and function. Fibroblast growth factors (FGFs) regulate a variety of events during brain development such as neuronal proliferation and differentiation, synaptic development and neurogenesis (Ornitz and Itoh, 2001; Thisse and Thisse, 2005; Umemori, 2009; Turner and Grose, 2010; Turner et al., 2012). Alteration in the FGF and FGF receptor signaling has been found in many neuropsychiatric diseases, including depression (Evans et al., 2004), altered social behavior (Scearce-Levie et al., 2008), seizures (Terauchi et al., 2010; Williams and Umemori, 2014), and intellectual disability (Williams and Umemori, 2014). Thus, understanding the precise roles of FGF signaling in brain development will shed light on the pathophysiology of such disorders and may provide clues for designing novel treatment strategies.

We have previously found that a subfamily of FGFs, Fibroblast Growth Factor 22 (FGF22), 7 and 10, acts as synaptic organizing molecules (Umemori et al., 2004). FGF22 is critical for the establishment of excitatory synapses in the developing brain, including the cerebellum, hippocampus and lateral geniculate nucleus (Umemori et al., 2004; Terauchi et al., 2010; Singh et al., 2012). Mice genetically lacking FGF22 (FGF22 null mice) show impaired excitatory synapse formation in the brain during development. The defects persist into adulthood: FGF22 null mice have fewer excitatory synapses later in life (Terauchi et al., 2010). Mechanistically, we proposed that FGF22 serves as a target (postsynaptic neuron)-derived presynaptic organizer in the hippocampus, because, using cultured neurons, we found: (i) FGF22 overexpression induced excitatory synapses on the FGF22 expressing neurons; and (ii) postsynaptic expression of FGF22 rescued impaired excitatory synapse formation in cultures prepared from FGF22 null mice (Terauchi et al., 2010). However, this idea has not been tested *in vivo*. Therefore, in this article, we ask whether FGF22 acts as a target-derived presynaptic organizer *in vivo* using mice in which FGF22 is selectively inactivated in CA3 pyramidal neurons, where FGF22 is highly expressed during development.

In addition to synapse development, FGF22 appears to be involved in the regulation of activity-dependent neurogenesis in the dentate gyrus, where neurogenesis continues throughout life. In wild-type mice, dentate neurogenesis has been shown to increase in response to seizure activity (Parent, 2007; Song et al., 2012; Lee and Umemori, 2013; Cho et al., 2015). In contrast, we found that FGF22 null mice do not show increased neurogenesis after seizure (Lee and Umemori, 2013). However, whether FGF22 is important for normal neurogenesis is not known. In this article, we ask whether FGF22 is involved in neurogenesis using FGF22 null mice. As CA3-derived FGF22 acts on the axons of dentate granule cells (DGCs), we also ask whether CA3-derived FGF22 plays a role in regulating neurogenesis using CA3-specific FGF22 knockout mice.

Finally, as a behavioral consequence of FGF22 inactivation, FGF22 null mice display depression-like behaviors such as increased passive stress-coping behavior and anhedonia (Williams et al., 2016). FGF22 null mice do not show any changes in anxiety-like behaviors, social cognition and motor phenotypes (Williams et al., 2016). These results suggest that FGF22 plays a unique role in affective behaviors, and FGF22 is a potential target for the development of novel antidepressant agents. In order to provide further insights into the molecular mechanisms underlying depression-like behaviors, in this article, we ask cell-type specific roles of FGF22 in the regulation of affective behavior using CA3-specific FGF22 knockout mice.

FGF22 is expressed by various neurons in the brain. In the hippocampus, FGF22 is highly expressed by CA3 pyramidal neurons as well as a subset of DGCs (Terauchi et al., 2010). In order to address the questions listed above, we utilized a conditional, cell-type specific FGF22 knockout mice. We generated CA3-specific FGF22 knockout (FGF22-CA3KO) mice and analyzed their synapse development, dentate neurogenesis, and affective behaviors. Here we show: (i) FGF22-CA3KO mice have reduced excitatory synapses formed onto CA3 pyramidal neurons; (ii) FGF22 null mice show decreased dentate neurogenesis throughout life; (iii) In contrast, FGF22-CA3KO mice does not show any changes in dentate neurogenesis; and (iv) FGF22-CA3KO mice exhibit increased passive stress coping behaviors and anhedonia, similarly to FGF22 null mice. These results suggest that CA3-derived FGF22 serves as a target-derived excitatory presynaptic organizer *in vivo* and contributes to the establishment of synaptic circuits involved in affective behavior.

## Materials and Methods

### Animals

**Figure 1:**
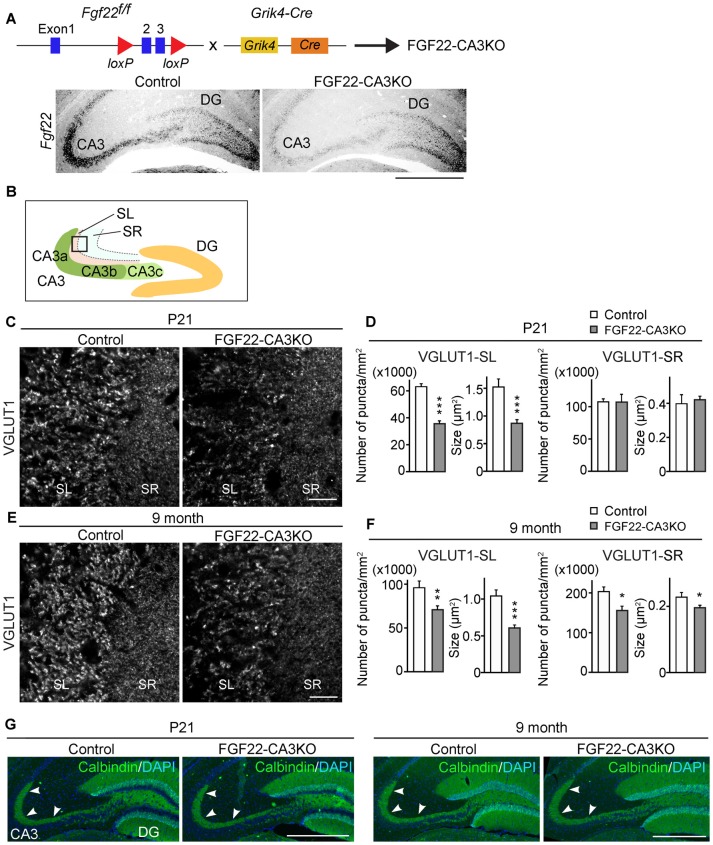
CA3-derived Fibroblast Growth Factor 22 (FGF22) is required for excitatory synapse formation in the hippocampal CA3 region. **(A)** (Top) Schematic diagram of the strategy to generate CA3-specific *Fgf22* knockout mice (FGF22-CA3KO mice). *Fgf22^flox/flox^* (*Fgf22^f/f^*) mice were crossed with mice carrying *Grik4*-promoter-driven Cre (*Grik4-Cre*). (Bottom) *In situ* hybridization for *Fgf22* mRNA with hippocampal sections from P9 wild-type littermates (Control) and FGF22-CA3KO mice (*Fgf22^f/f^*::*Grik4-Cre*). *Fgf22* expression is specifically eliminated from CA3 pyramidal neurons in FGF22-CA3KO mice. **(B)** Images in **(C,E)** were taken from the boxed area. Analysis was performed from images taken in CA3a and CA3b. **(C,D)** Hippocampal sections from Control and FGF22-CA3KO mice at P21 were immunostained for vesicular glutamate transporter 1 (VGLUT1). **(C)** Representative pictures from the CA3 region. SL, stratum lucidum; SR, stratum radiatum. Density (number/mm^2^) and size (μm^2^) of VGLUT1 puncta were quantified and shown in **(D)**. **(E,F)** Hippocampal sections from adult Control and FGF22-CA3KO mice (9 months of age) were immunostained for VGLUT1. **(E)** Representative pictures from the CA3 region. Density and size of VGLUT1 puncta were quantified and shown in **(F)**. FGF22-CA3KO mice show decreased VGLUT1 puncta in the CA3 SL layer at P21 and in SL and SR layers in adults. Bars indicate mean ± SEM. **(G)** DGC axon targeting is not impaired in FGF22-CA3KO mice. Hippocampal sections were immunostained for calbindin to visualize DGC axons (arrowheads). Data are from **(C)** 10–17 sections from five mice and **(E)** 13–22 fields in 5–7 sections from three mice. Significant difference from wild-type mice at *p < 0.05, **p < 0.01, and ***p < 0.001 by Student’s *t*-test. Scale bars, **(A,G)** 500 μm, **(C,E)** 20 μm.

*Fgf22*^−/−^ mice (FGF22 null mice) were described previously (Terauchi et al., 2010). *Fgf22*^−/−^ mice were backcrossed with C57/BL6J (Jackson Laboratories, Bar Harbor, ME, USA) for more than 15 generations. *Fgf22^flox/flox^* mice (*Fgf22^tm1a(EUCOMM)Hmgu^*) were from EUCOMM (Terauchi et al., 2016). Grik4-Cre mice were from Jackson (Nakazawa et al., 2002). *Fgf22^flox/flox^* mice were mated with Grik4-Cre mice to generate CA3-specific FGF22 knockout mice (FGF22-CA3KO mice; see Figure 1A). Both males and females were used in our study. The numbers of animals used in the behavioral studies are shown in Table 1 and figure legends. The numbers of animals used in the histological analysis are shown in figure legends. All animal care and use was in accordance with the institutional guidelines and approved by the Institutional Animal Care and Use Committees at Boston Children’s Hospital.

**Table 1:** List of the numbers and ages of animals used in the behavioral tests.

	Forced swim		Sucrose preference	
	*N*	Age (month)	*N*	Age (month)
Control				
Total	38	5.77 ± 0.38	25	7.87 ± 0.57
Females	21	5.85 ± 0.51	14	8.61 ± 0.79
Males	17	5.33 ± 0.55	11	6.93 ± 0.76
FGF22-CA3KO				
Total	29	5.26 ± 0.36	21	6.86 ± 0.54
Females	16	5.41 ± 0.55	11	7.55 ± 0.79
Males	13	5.02 ± 0.43	10	6.09 ± 0.70

### Immunostaining and Antibodies

Mice were transcardially perfused with PBS followed by 4% paraformaldehyde (PFA; Electron Microscopy Sciences, Hatfield, PA, USA) in PBS. Brains were dissected, further fixed in 4% PFA in PBS for overnight, transferred in 30% sucrose in PBS, and frozen in Neg-50 Frozen Section Medium (Richard Alan Scientific, Kalamazoo, MI, USA). Coronal sections were prepared on a cryostat (16 μm thick), and processed for staining. The sections were blocked in 2% BSA (Fraction V, Sigma-Aldrich, St. Louis, MO, USA), 2% normal goat serum (Sigma-Aldrich), and 0.1% TritonX-100 (Sigma-Aldrich) for 30 min at room temperature followed by incubation with primary antibodies for overnight at 4°C or for 2 h at room temperature. The sections were washed with PBS three times, and secondary antibodies were applied for 1 h at room temperature. The sections were washed with PBS three times again, and the slides were mounted in glycerol with 0.5% p-phenylenediamine (Sigma-Aldrich). Dilutions and sources of primary antibodies used are: anti-doublecortin (DCX; 1:500, ab18723, Abcam, Cambridge, MA, USA), anti-Prox1 (1:500, MAB5652, EMD Millipore, Burlington, MA, USA), anti-vesicular glutamate transporter 1 (anti-VGLUT1; 1:5000, AB5905, EMD Millipore), anti-vesicular GABA transporter (anti-VGAT; 1:1500, 131003, Synaptic Systems, Goettingen, Germany), anti-calbindin (1:500, Calbindin-Go-Af1040, Frontier Institute, Hokkaido, Japan). Secondary antibodies used are (1:500 dilutions): Alexa 488-conjugated goat anti-guinea pig IgG (A-11073, Invitrogen, Carlsbad, CA, USA), Alexa 568-conjugated goat anti-guinea pig IgG (A-11075, Invitrogen), Alexa 488-conjugated goat anti-rabbit IgG (A-11034, Invitrogen), Alexa 568-conjugated goat anti-rabbit IgG (A-11036, Invitrogen), Alexa 488-conjugated goat anti-mouse IgG1 (A-21121, Invitrogen), and Alexa 594-conjugated donkey anti-goat IgG (705–585–147, Jackson ImmunoResearch, West Grove, PA, USA). DAPI (Sigma-Aldrich) was added to each section as a nuclear stain.

### *In Situ* Hybridization

*In situ* hybridization was performed as described (Schaeren-Wiemers and Gerfin-Moser, 1993; Terauchi et al., 2010). Digoxigenin-labeled cRNA probes were generated by *in vitro* transcription using DIG RNA labeling mix (Roche, Basel, Switzerland). The probe for *Fgf22* was generated from the cording region of the mouse *Fgf22* cDNA. *In situ* images were taken with a digital camera (Alpha 5100, Sony, Tokyo, Japan) attached to an Olympus BX63 upright microscope (Olympus, Tokyo, Japan) under bright-field optics with 10× objective lenses.

### Imaging and Analysis

Fluorescent images were taken on epi-fluorescence microscopes (Olympus BX61 and BX63). Twelve-bit images were acquired using 20× objective lenses with an F-View II CCD camera (Olympus Soft Imaging Solutions, Muenster, Germany) or an XM10 Monochrome camera (Olympus) at 1376 × 1032 (Olympus BX61) or 1376 × 1038 (Olympus BX63) pixel resolution. Images were taken at the best-focus position, where the staining signals were brightest in the section.

Synapse formation in the CA3 region was assessed using immunostaining for VGLUT1 and VGAT. Images were taken from the CA3a and CA3b regions of CA3 (see Figure 1B). The size and density of stained puncta were quantified and analyzed using MetaMorph software (Molecular Devices, Sunnyvale, CA, USA). The staining intensity in the fimbria, a myelinated tract of axons located in the medial region of CA3, was calculated and used for background subtraction from each image. Neurogenesis within the DGC layer was assessed using immunostaining for DCX, a marker for immature neurons, and Prox1, a marker for DGCs. The number of DCX-positive cells was divided by the number of Prox1-positive DGCs or that of DAPI-positive DGCs. Quantification was done from both the upper and lower blades of the DGC layer.

### Behavioral Tests

#### Forced Swim Test

Mice were allowed to adapt to the testing room environment at least for 30 min before the test. Each animal was then placed into a 4 L beaker with 2.75 L of tepid water (21–23°C) for 6 min, and their behavior was recorded using a video camera (HDC-SD60, Panasonic, Osaka, Japan) mounted on the same level as the base of the beaker. Video recordings were reviewed and scored by a trained observer blinded to genotype. Animals were assessed for the total duration of floating during the last 4 min and the latency to start floating for more than 3 s in the water. Mice were judged to be floating when making only the movements necessary to keep their heads above water.

#### Sucrose Preference Test

Sucrose preference was assessed using a two-bottle choice experiment: one bottle containing water and the other containing 2% (v/v) sucrose for 5 days. One day before the test, mice were singly housed in their housing room and given a bottle filled with 2% sucrose to experience sweet taste. On the morning of day 1, a sucrose bottle and a water bottle were weighed and placed in the cage. Twenty-four hours later, the bottles were weighed and the bottle positions were switched, to control for any side position preferences in the mice. The bottle positions were switched every 24 h during the 5-days test, weighing the bottles each day before switching. Data are presented as the amount of sucrose consumed as a percentage of total liquid consumed over the testing period.

### Statistical Analysis

Data were prepared and analyzed using Excel or GraphPad Prism 7. The statistical tests performed were two-tailed Student’s *t*-test as indicated in the figure legend. No data points were excluded from any experiments. The results were considered significant when *p* < 0.05 (denoted in all graphs as follows: *p < 0.05; **p < 0.01, ***p < 0.001).

## Results

### CA3-derived FGF22 Is Critical for Excitatory Presynaptic Differentiation in the Hippocampal CA3 Region

FGF22 plays important roles for excitatory synapse formation in the mammalian brain, including the hippocampus, cerebellum and lateral geniculate nucleus (Umemori et al., 2004; Terauchi et al., 2010; Singh et al., 2012). Mice genetically lacking FGF22 (FGF22 null mice) have decreased excitatory presynaptic terminals in the brain. In the hippocampus, FGF22 is highly expressed by hippocampal CA3 pyramidal neurons but also expressed by a subset of dentate granule cells (DGCs; Terauchi et al., 2010). Aiming at understanding the cell type-specific role of FGF22, we generated CA3-specific FGF22 knockout mice (FGF22-CA3KO mice) using *Fgf22^flox/flox^* mice (*Fgf22^f/f^*; EUCOMM) crossed with *Grik4-Cre* mice (Figure 1A). *Grik4-Cre* mice express Cre in nearly 100% of CA3 pyramidal neurons (Nakazawa et al., 2002). In other regions in the brain, Cre is only expressed in a very small subset (less than 10%) of neurons, if any (Nakazawa et al., 2002). We first confirmed the CA3-specific inactivation of *Fgf22* in FGF22-CA3KO mice by performing *in situ* hybridization at P9, during synapse formation (Figure 1A). In wild-type control mice, *Fgf22* was highly expressed in CA3 pyramidal neurons and at a lower level, in some of DGCs. In *Fgf22^f/f^*::*Grik4-Cre* mice, *Fgf22* expression in CA3 pyramidal neurons was almost completely eliminated, while it was maintained in DGCs. These results demonstrate that *Fgf22^f/f^*::*Grik4-Cre* mice are indeed CA3-specific FGF22 knockout mice. Since *Fgf22* expression in control mice was highest in the CA3a and CA3b subregions (Figures 1A,B), we focused on these subregions for the analysis of synapses. In order to investigate whether CA3-derived FGF22 is necessary for excitatory presynaptic differentiation in CA3, we evaluated the clustering of excitatory synaptic vesicles by staining for VGLUT1, which is on the excitatory synaptic vesicles. We found that at P21, FGF22-CA3 KO mice showed a significant decrease in the number and size of VGLUT1 puncta in the CA3 stratum lucidum (SL) region, where the axons of DGCs form excitatory synapses with CA3 pyramidal neurons (Figures 1C,D), without apparently affecting the DGC axon targeting (assessed by calbindin staining; Figure 1G). In adult FGF22-CA3KO mice (9 months of age), clustering of VGLUT1 was reduced in the SL as well as the stratum radiatum (SR) region, where CA3 axons form excitatory synapses onto CA3 pyramidal neurons (Figures 1E,F). These results indicate that FGF22, derived from CA3 pyramidal neurons, is critical for the development of excitatory presynaptic terminals formed onto CA3 pyramidal neurons. Our results demonstrate that FGF22 is a target-derived presynaptic organizer in the mammalian hippocampus *in vivo*.

### CA3-derived FGF22 Does Not Regulate Inhibitory Presynaptic Differentiation in the Hippocampal CA3 Region

**Figure 2:**
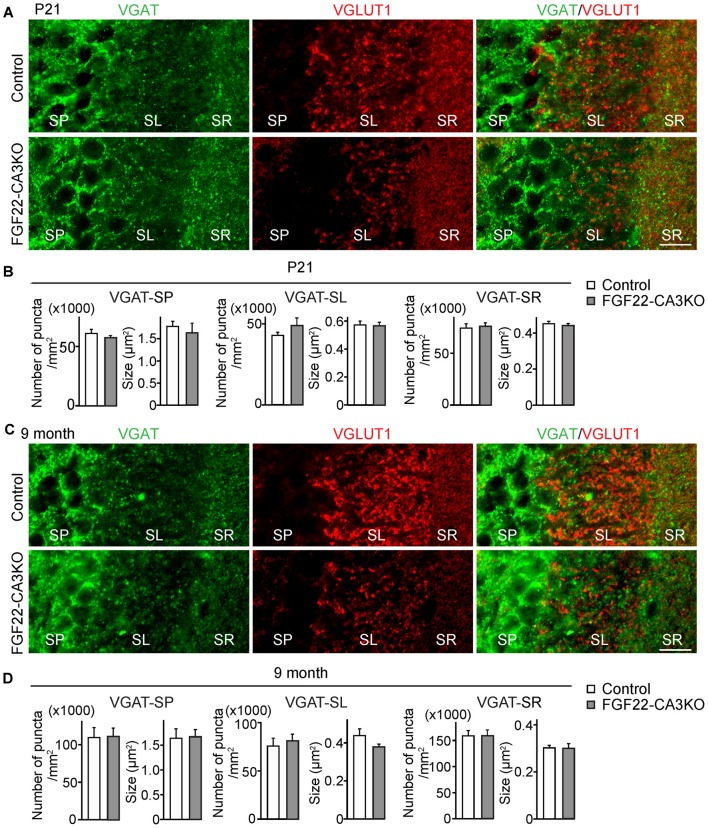
CA3-specific FGF22 deletion does not alter inhibitory synapse formation in CA3. Hippocampal sections from Control and FGF22-CA3KO mice were immunostained for vesicular GABA transporter (VGAT) and VGLUT1. **(A)** Representative pictures from CA3 at P21. SP, stratum pyramidale; SL, stratum lucidum; SR, stratum radiatum. **(B)** Quantification of the density and size of VGAT puncta in CA3 at P21. **(C)** Representative pictures from CA3 in adults (9 months of age). **(D)** Quantification of the density and size of VGAT puncta in CA3 in adults. There is no significant difference in inhibitory synapse formation, assessed by VGAT accumulation, between Control and FGF22-CA3KO mice at P21 and in adults. Bars indicate mean ± SEM. Data are from **(B)** 24 fields in eight sections from three mice and **(D)** 18 fields in six sections from three mice. Scale bars, 20 μm.

We next asked whether CA3-derived FGF22 is necessary for inhibitory presynaptic differentiation in CA3. For this, we evaluated the clustering of inhibitory synaptic vesicles by staining for VGAT. Inhibitory presynaptic differentiation was not impaired in the stratum pyramidale (SP), SL and SR layers of the CA3 region of FGF22-CA3KO mice at P21 (Figures 2A,B) and in adults (9 months of age, Figures 2C,D). Thus, CA3-derived FGF22 is an excitatory synapse-specific presynaptic organizer in the hippocampus.

### FGF22 Is Involved in Neurogenesis in the Dentate Gyrus Throughout Life

**Figure 3:**
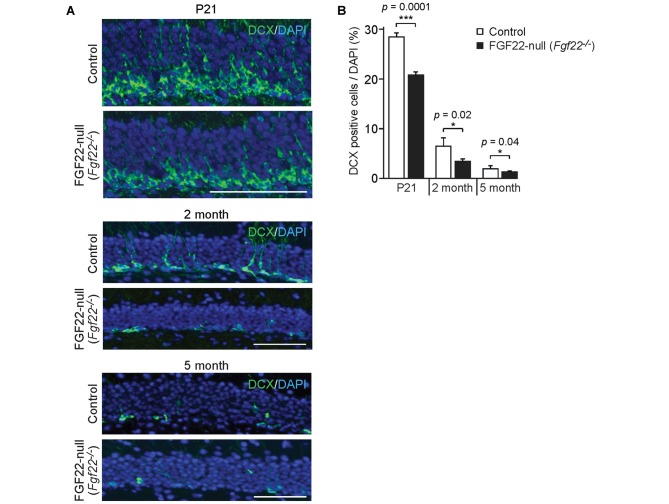
FGF22 null mice (*Fgf22*^−/−^) show reduced dentate neurogenesis throughout life. Region-matched hippocampal sections from wild-type littermates (Control) and FGF22 null mice (*Fgf22*^−/−^) at P21, 2 months of age and 5 months of age were immunostained for doublecortin (DCX), a marker for immature neurons. **(A)** Representative pictures of DCX-positive cells (green) with DAPI (blue) in the dentate granule cell (DGC) layer of Control and FGF22 null mice. **(B)** Quantification of the percentage of DCX-positive cells in the DGC layer. Dentate neurogenesis, assessed by the number of DCX-positive cells in DGCs, was significantly decreased in FGF22 null mice compared to wild-type mice at all the age points examined. Bars indicate mean ± SEM. Data are from nine sections from three mice (P21) and 4–6 sections from three mice (2 and 5 months of age). Scale bars, 100 μm.

In the hippocampus, the dentate gyrus undergoes continuous neurogenesis throughout life, and the newly born DGCs are integrated into pre-existing neuronal circuits. Because various growth factors control both developmental and adult neurogenesis in the brain (Zhao et al., 2007; Haan et al., 2013; Oliveira et al., 2013; Vivar et al., 2013; Woodbury and Ikezu, 2014; Kang and Hebert, 2015), we next asked whether FGF22 plays a role in dentate neurogenesis in the hippocampus. We first examined neurogenesis in FGF22 null mice. We assessed neurogenesis in the DGC layer by staining hippocampal sections for DCX, a marker of immature neurons (Francis et al., 1999; Gleeson et al., 1999; Abrous et al., 2005). FGF22 null mice had a significantly lower number of DCX-positive cells in the DGC layer relative to wild-type mice at P21 (Figures 3A,B), suggesting that neurogenesis is decreased in the developing hippocampus in the absence of FGF22. Decreased neurogenesis was also detected in FGF22 null mice at older ages (2 and 5 months of age; Figures 3A,B). These results indicate that FGF22 contributes to dentate neurogenesis throughout life.

### CA3-derived FGF22 Does Not Contribute to Neurogenesis in the Dentate Gyrus

**Figure 4:**
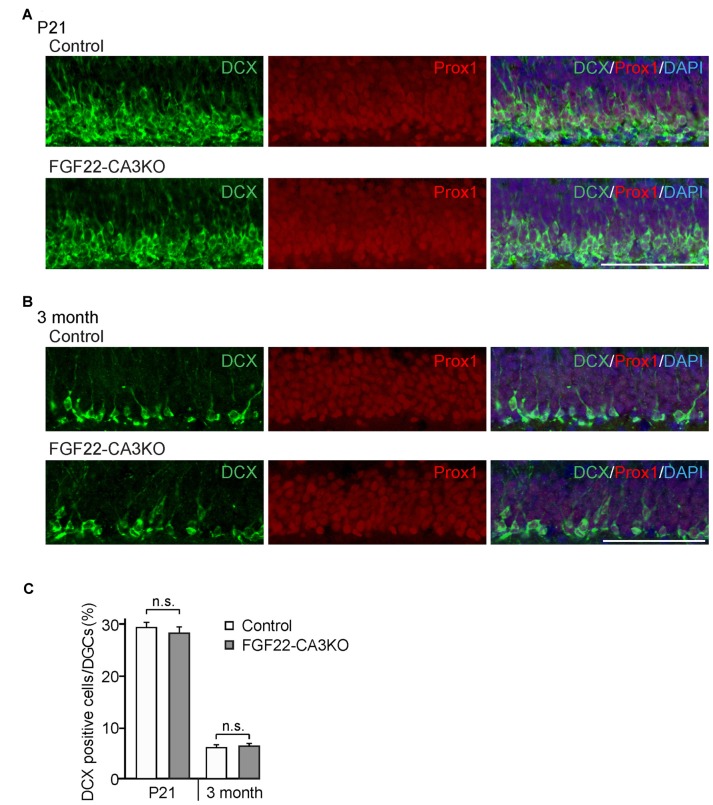
Dentate neurogenesis is not affected in CA3-specific FGF22 knockout mice. Region-matched coronal brain sections from Control and FGF22-CA3KO mice were immunostained for DCX and Prox1, a marker for DGCs. **(A,B)** Representative pictures of DCX-positive cells (green) and Prox1-positive cells (red) in the DGC layer of Control and FGF22-CA3KO mice at P21 **(A)** and in adults (3 months of age; **B**). **(C)** Quantification of the percentages of DCX-positive cells in Prox1-positive DGCs. Dentate neurogenesis, assessed by the number of DCX-positive cells, did not differ between Control and FGF22-CA3KO mice. Bars indicate mean ± SEM. Data are from eight sections from three mice (both P21 and 3 months of age). Scale bars, 100 μm.

How does FGF22 regulate dentate neurogenesis? One possibility is that CA3-derived FGF22 sends retrograde signals to DGCs and influences dentate neurogenesis. We have previously shown that, using microfluidic chambers, application of FGF22 to the axons of DGCs induces insulin-like growth factor 2 (IGF2) in the cell body of DGCs (Terauchi et al., 2016). Hence, one hypothesis is that CA3-derived FGF22 regulates gene expression in DGCs, which may influence dentate neurogenesis. On the other hand, FGF22 is also expressed by a subset of DGCs (18.5% ± 1.24%) as well as CA3 pyramidal neurons, so it is possible that DGC-derived FGF22 has a main role on dentate neurogenesis. Using FGF22-CA3KO mice, we investigated whether CA3-derived FGF22 is involved in dentate neurogenesis. Dentate neurogenesis, assessed by the number of DCX-positive cells in DGCs, is unchanged in FGF22-CA3KO mice at P21 (Figures 4A,C) and in adults (3 months of age, Figures 4B,C). The number of Prox1-positive DGCs was also not changed in FGF22-CA3KO mice (P21: Control 13281.56 ± 263.61 cells/mm^2^, FGF22-CA3KO 13094.29 ± 259.32 cells/mm^2^; 9-month-old: Control 11901.01 ± 180.17 cells/mm^2^, FGF22-CA3KO 11940.50 ± 164.63 cells/mm^2^). These results suggest that CA3-derived FGF22 does not regulate dentate neurogenesis.

### FGF22-CA3KO Mice Show Depression-Like Behaviors

**Figure 5:**
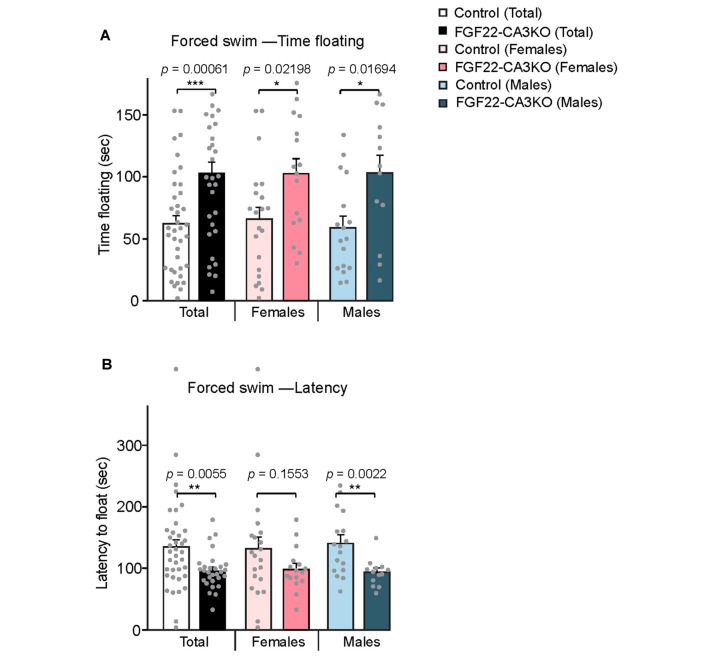
CA3-specific FGF22 knockout mice display increased passive stress-coping responses in the forced swim test.** (A)** Time floating during the 4-min forced swim test for Control littermates and FGF22-CA3KO mice. Data separated by the gender are also shown. FGF22-CA3KO mice, both females and males, spent significantly more time floating than control mice. **(B)** Latency to float for Control littermates and FGF22-CA3KO mice. FGF22-CA3KO mice start floating more quickly. Bars indicate mean ± SEM. The numbers of mice used were: 21 females and 17 males for Control, and 16 females and 13 males for FGF22-CA3KO. Significant difference from controls at *p < 0.05, **p < 0.01 and ***p < 0.001 by Student’s *t*-test.

The hippocampus is implicated in mood, anxiety, and learning and memory (Femenia et al., 2012; Bannerman et al., 2014; Tovote et al., 2015). FGF22 null mice show reduced excitatory synapse formation (Terauchi et al., 2010) and dentate neurogenesis (Figure 3) in the hippocampus. As a behavioral consequence, FGF22 null mice show depression-like behaviors such as increased passive stress-coping behavior and anhedonia (Williams et al., 2016). FGF22 null mice display normal motor, anxiety and social cognitive tests, indicating a role of FGF22 specifically in affective behaviors. Using FGF22-CA3KO mice, we asked whether CA3-derived FGF22 plays a role in regulating affective behavior. The forced swim test is one of the commonly used rodent behavioral tests to measure coping strategy to an acute inescapable stress (Kitada et al., 1981). It is often used as a behavioral screen for antidepressant drugs and evaluating the efficacy of the drugs. In the forced swim test, we found that both female and male FGF22-CA3KO mice spent significantly more time floating than control littermates (Figure 5A). In addition, FGF22-CA3KO mice displayed a shorter latency to float than control littermates (Figure 5B; when females and males were separately analyzed, males showed more significant differences). These results show that FGF22-CA3KO mice display increased passive stress-coping phenotypes, suggesting that CA3-derived FGF22 plays important roles in regulating stress-coping behavior.

**Figure 6:**
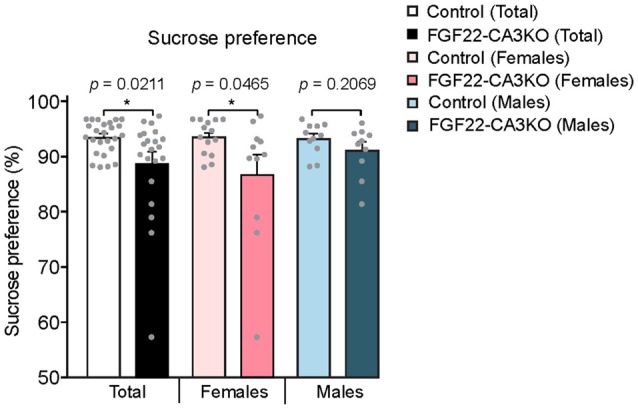
CA3-specific FGF22 knockout mice display anhedonia. Sucrose preference test with Control littermates and FGF22-CA3KO mice. Data shown are the percentage of sucrose consumed relative to total liquid consumed. FGF22-CA3KO mice drink less sucrose than Control littermates. Bars indicate mean ± SEM. The numbers of mice used were: 14 females and 11 males for Control, and 11 females and 10 males for FGF22-CA3KO. Significant difference from controls at *p < 0.05 by Student’s *t*-test.

We next tested whether FGF22-CA3KO mice display anhedonia, the failure to engage in pleasurable activity, which is a hallmark of depression. The sucrose preference test is a reward-based behavioral test to assess preference for sucrose-sweetened water over regular water and used as an indicator of anhedonia (Powell et al., 2012). Typically, mice prefer to drink sucrose with a high ratio, but mice that display anhedonia show less preference to sucrose. We found that FGF22-CA3 KO mice demonstrated a significantly less preference to sucrose over water than control littermates (Figure 6), consistent with anhedonia. When females and males were separately analyzed, females showed more significant changes.

Taken together, these behavioral tests suggest that FGF22-CA3KO mice exhibit behavioral changes consistent with depression-like behaviors and that there are some sex differences in their phenotype.

## Discussion

FGF22 plays important roles in brain development and function (Umemori et al., 2004; Terauchi et al., 2010; Singh et al., 2012; Williams et al., 2016). In this article, we investigated the cell type-specific roles of FGF22 in the hippocampus. Using CA3-specific FGF22 knockout mice (FGF22-CA3KO mice) and FGF22 null mice, we showed: (i) CA3-derived FGF22 acts as a target-derived presynaptic organizer at excitatory synapses *in vivo*; (ii) FGF22 regulates dentate neurogenesis, but CA3-derived FGF22 does not play such a role; and (iii) Inactivation of FGF22 selectively in CA3 pyramidal neurons is sufficient for the mice (FGF22-CA3KO) to display increased stress coping behaviors and anhedonia, similarly to FGF22 null mice. These results indicate that CA3-derived FGF22 plays critical roles in local synapse formation and the regulation of affective behavior. Our results reveal cell-type specific roles of FGF signaling in brain development and function.

### FGF22 as a Target-derived Presynaptic Organizer *in Vivo*

In cultured neurons, FGF22 promotes synaptic vesicle accumulation at the excitatory nerve terminals (Umemori et al., 2004; Terauchi et al., 2010). In FGF22 null mice, synaptic vesicles fail to cluster at excitatory synapses in the CA3 region of the hippocampus (Terauchi et al., 2010). These results indicate that FGF22 organizes excitatory presynaptic terminals. Using cultured neurons, we had proposed that FGF22 acts as a target-derived presynaptic organizer, because: (i) overexpression of FGF22 in cultured neurons increased excitatory synapses formed onto FGF22-transfected neurons; and (ii) postsynaptic expression of FGF22 rescued the presynaptic defects in FGF22-deficient neurons (Terauchi et al., 2010). However, since cultured neurons lack precise spatial information, it is important to test whether FGF22 really acts as a local, target-derived factor *in vivo*. Here, utilizing CA3-specific FGF22 knockout (FGF22-CA3KO) mice, we showed that FGF22 indeed acts as a target-derived excitatory presynaptic organizer *in vivo*: FGF22-CA3KO mice showed excitatory, and not inhibitory, presynaptic defects in CA3 (Figures 1, 2). There was one difference between FGF22-CA3KO and FGF22 null mice: the synaptic defect in the CA3 SR layer of FGF22 null mice was apparent at P21, but it was not apparent until adults in FGF22KO-CA3KO mice. This may be due to a delayed onset of Cre expression in *Grik4-Cre* mice (Cre starts to express from P4 and gradually increases during development; Nakazawa et al., 2002; Allen Mouse Brain Atlas found at http://mouse.brain-map.org/).

### The Role of FGF22 in Dentate Neurogenesis

We found that FGF22 is involved in dentate neurogenesis throughout life (Figure 3). Since FGF22 is expressed by CA3 pyramidal neurons as well as a subset of DGCs (Terauchi et al., 2010; Figure 1A), FGF22 from either cell could contribute to dentate neurogenesis. FGF22 from DGCs may directly regulate dentate neurogenesis. Indeed, deletion or activation of FGF receptors in neural precursor cells modifies maintenance of SGZ stem cells and induction of neurogenesis (Kang and Hebert, 2015). In addition, FGF22 from CA3 neurons may induce gene expression in the presynaptic DGCs, and induced genes may influence neurogenesis. As for the latter possibility, we have previously shown that FGF22 signaling induces IGF2 in DGCs (Terauchi et al., 2016). IGF2 is known to regulate neurogenesis, so it is possible that CA3-derived FGF22 may regulate dentate neurogenesis through the expression of IGF2. Here we tested this possibility using FGF22-CA3KO mice. We found that FGF22-CA3KO mice did not show changes in dentate neurogenesis (Figure 4), indicating that CA3-derived FGF22 is not necessary for dentate neurogenesis. This suggests that FGF22 from other cells, possibly DGCs, contributes to dentate neurogenesis.

Neurogenesis is also dependent on neuronal activity. For example, enriched environment enhances the survival of newborn neurons (Leuner et al., 2006; Drapeau et al., 2007; Sisti et al., 2007); physical exercises, such as wheel running and forced treadmill, increase the proliferation of precursor neurons (van Praag et al., 1999) and LTP induction in the hippocampus increases proliferation of dentate progenitor cells and helps survival of new born DGCs (Bruel-Jungerman et al., 2006). Interestingly, we have previously found that FGF22 is important for seizure-induced dentate neurogenesis: FGF22 null mice do not display increased neurogenesis in response to seizures (Lee and Umemori, 2013). How FGF22 regulates normal and activity-dependent dentate neurogenesis is an important future question to address.

### Implication of CA3-derived FGF22 in Affective Behavior

FGF22 null mice display a depression-like phenotype: increased passive stress coping behavior and anhedonia (Williams et al., 2016). FGF22 null mice do not display defects in motor and exploratory behaviors, anxiety-like behaviors and social behaviors or memory. These results suggest that FGF22 has a unique role in affective behaviors. Here, using FGF22-CA3KO mice, we showed that loss of FGF22 in CA3 pyramidal neurons was enough to affect affective behaviors: FGF22-CA3KO mice showed increased floating time and decreased latency to float in the forced swim test, and decreased preference for sucrose in the sucrose preference test (Figures 5, 6). Our results also suggest that there are some differences between females and males in the behavioral tests. It would be interesting to further examine whether and how FGF22 acts differently in females and males.

In summary, these results show a cell-type specific role of FGF22 in affective behavior. Our results suggest that FGF22-dependent synapse development in CA3, and not dentate neurogenesis, may be regulating affective behavior. Adult dentate neurogenesis is proposed to contribute to a depression-like behavior (Lee et al., 2013; Anacker and Hen, 2017), while some studies showed that mice lacking adult neurogenesis did not display abnormal stress-coping behaviors (Iascone et al., 2013; Jedynak et al., 2014). It is likely that there are various molecular and pathophysiological changes that contribute to depression-like behaviors, and we propose that synapse development mediated by CA3-derived FGF22 is one such underlying mechanism. Our study raises a possibility to target FGF22 in CA3 as a possible treatment of certain aspects of depression.

## Author Contributions

AT and HU designed the experiments and wrote the manuscript. AT, EG and JW performed the experiments. HU secured funding and supervised the project. All authors commented on the manuscript.

## Conflict of Interest Statement

The authors declare that the research was conducted in the absence of any commercial or financial relationships that could be construed as a potential conflict of interest.
